# Notes from Past Meetings of the Lippe Society

**Published:** 1889-06

**Authors:** George H. Clark


### NOTES FROM PAST MEETINGS OF THE LIPPE SOCIETY.

MESSRS. EDITORS :—In glancing over the minutes of some past meetings of the Lippe Society, I am constrained to compare the proper method of showing the value of our pathogeneses, and the way adopted by some self-styled teachers of materia medica.

The following remarks of Dr. Lippe are taken from the minutes of a meeting held February 17th, 1880. Although they contain nothing new, they are of permanent value, and I am sure you cannot give space to anything of more worth : "Hahnemann was the first to prove Aconite. In reading this proving we should first read the preface. Hahnemann then gives us what we may properly call the key-note of Aconite. This, however, is not the modern key-note. Hahnemann's key-notes are very few. The last one is the most important.

"When Aconite is prescribed, the mental symptoms must be present, else that remedy is not indicated. The key-notes are: Irresistible restlessness, agonizing tossing about, thirst, and fever. In fevers (pathological) the above symptoms must be present.

"Why do the mongrels give Aconite in large doses? Because they are looking for pathological conditions.

"Another mental symptom is to be found in the effects of fright and anger, and here it is a great remedy. Thus, for suppressed menses from fright and anger, Acon. is the remedy. The pathological people do not believe that fright and anger will stop the menstrual flow. Hence they miss excellent cures. Where the feet get wet, and there is suppression of menses as a consequence, Puls. is the remedy.

"In the winter of 1838, a young girl was baptized in a stream where the ice had to be broken for the purpose; suppression of menses followed, and a condition giving a perfect picture of Acon. came on. Notwithstanding the clergyman's saying that no medicine was needed for such a case, I gave Acon., which soon brought about a normal flow.

"The pathological gentlemen do not believe in Acon. for the effects of fright, because they never read Hahnemann's preface to the proving.

"We never see Acon. indicated in typhoid fever. A young lady had been suffering with fever. Her mother gave her Acon. as directed by the domestic books, and the fever disappeared. The next day the fever reappeared and she was again given Acon. Thus it went on for a week, when I was called and found her advanced in typhoid fever. The Acon. was not indicated, and had retarded the cure.

"If the other symptoms correspond, and the mental symptoms are present, then you can successfully give Acon., and only then. The Acon. restlessness is agonizing. The patient does not *tumble*, he *tosses* about.

"We have another kind of restlessness in which the patient tumbles about like a kitten. He goes so fast and so constantly that we cannot get a chance to talk to or question him. This is Apis.

"Arum tri. has restlessness with picking of the lips till they bleed. In such cases Arum will cure both symptoms.

"In Rhus tox. cases the patient is restless and turns to a new position. He remains quiet for a few minutes and then changes. The Puls. patient cannot lie quiet, but must turn to a new position constantly. The very idea of moving is terrible, yet he *must* move; but he is not relieved by the change of position. The Rhus patient, on the other hand, does find relief for a few minutes in the new position.

"All that we know of Acon. is found in the *Materia Medica Pura*—thirst, fever, and the agonizing tossing about. That is the whole story.

"Acon. is useful only in the worst kind of acute cases. I do not touch my Acon. vial once in six months. It is useful in cases of hypertrophy of the heart. The patient cannot sleep because he is so restless and in such fear. This drug will not cure the heart trouble, but it will make the patient sleep regularly, sometimes for months. In labor, where there is the agonizing tossing about, with fear that she cannot stand the pain, Acon. is indicated. As I have said, I seldom find it indicated, but when it is given its results are wonderful.

"In the present epidemic of measles, Acon. is frequently indicated. In some cases Puls. follows.

"In brain diseases it is at times indicated. In children, when they are better from being carried about, Cham. is the remedy. Similar to Cham. are Ant. tart. and Ignatia. Where Bell. is indicated, the child may desire to be carried about, but the headache gets worse from the motion. The child may not be able to mention this fact, and it may escape observation, and we may then wrongfully prescribe Cham. To give Cham. to every child having a desire to be carried about is to make serious blunders in many cases. In the Bell. case the headache is worse from lying down. By noticing this we will not mistake and prescribe Cham.

"The Acon. headache is better from lying down. Acon. has very little that can be compared with any other remedy.

"There is some little similarity between Acon. and Bell. Acon. has cough which is short, as we find it in measles. A short, hacking, continuous cough. There is pain in the region of the liver, also stitches. The mental symptoms occur with the Acon. cough. The child will not lie quiet. If he does remain quiet Scilla is the remedy.

"It is a great mistake to give Acon. in measles merely because there is an eruption with fever.

"I recollect giving Puls. to a case of measles, thus bringing out the eruption. Vomiting then set in. The child vomited all day, but I decided to give it nothing else. Next diarrhœa appeared. I left a dose of Sulph. with the mother, to give in case the diarrhœa did not disappear by midnight. Still, I felt certain the diarrhœa would soon cease, and it did. The Puls. brought out the eruption. Then came the symptom, involuntary stool while urinating. This symptom occurs in Mur. acid and Sulph.

"I have cured more cases of croup with Bell. than with any other remedy."

From these few notes one can see how a master taught materia medica.

At a meeting held on March 16th, 1880, Dr. Lippe, in connection with a statement by Dr. C. Carleton Smith, of a case of fistula nine inches in length, in the knee joint, subsequent to a maltreated attack of typhoid fever, said that Lycopodium was a great remedy in the after effects of typhoid fever.

Dr. Lippe then described a case of pneumonia in illustration of the repetition of the dose. The patient was given Sulphur, which did not act promptly, and had to be repeated for three or four days. It caused him to perspire profusely; but as soon as he ceased to take the Sulph. the perspiration ceased. He had never perspired before—not even while living in the West Indies. Dr. Lippe was ready to give him up as a hopeless case, when suddenly the old man set to coughing. It was an incessant cough, day and night, and he stormed and swore at the doctor for not relieving him. It was not thought advisable to stop the cough, as it was necessary to his recovery, and he was given no medicine. The cough soon disappeared. Then a severe diarrhœa set in. Still no medicine. He shortly got well.

Dr. Lee thought that it was always dangerous to stop a cough in serious lung troubles.

Dr. Clark related the case of a woman whose father, mother, and brother died of tuberculosis. The woman had been running down for two years. With a violent cough, she had many other serious symptoms, which were conquered by appropriate remedies. Her cough still continued, and she was constantly begging for something to stop her cough, notwithstanding her general condition was all that could be desired. She was warned against having the cough stopped.

She was under Dr. Clark's treatment for three years, continuing well, with the exception of the cough. While visiting in Brooklyn she contracted a heavy cold. She was then treated by a so-called homœopathic physician, who gave her material doses of Opium. Much to her delight her cough stopped. In less than two weeks afterward she was in her grave.

Dr. Lippe said he had cut his eye-teeth many years ago in a case of typhoid fever, in a drunken Irishman. On the seventh day after the delirium was over his nose began to bleed. Without giving any remedy than that given previous to this symptom, he waited for fourteen days, the bleeding continuing for that time, and the man rapidly recovered.

Dr. C. Carleton Smith remarked that he could never see the use of prescribing medicine every two hours. It is a perfectly arbitrary period of time, and there does not seem to be any sense in it.

Dr. Lippe—It is a common thing to meet with so-called homœopathists who give several remedies at a time, in repeated doses. If you give Bell. in more than one dose, you will be sure to witness an increase of fever the next day at four o'clock.

Dr. Lee reported a case where there was pain in the right arm ; must lie on the arm. The patient complained of being unable to raise the arm to the head. There was no particular reason why she should raise the arm to the head, but this spontaneous expression of the symptom led him to give a dose of Sanguinaria. It produced no effect, whereupon he gave it in water, and the patient was relieved in that arm, and the pain then appeared slightly in the left. This would seem to show the advantage of many doses.

He asked the question as to whether he should have let the one dose have more time to act.

Dr. Lippe answered that he probably did right ; that the repetition of the dose was necessary in many patients with feeble vitality ; that Dr. Lee's patient was a poor subject, and that there would be much trouble in curing her. In such cases it is necessary to repeat the remedy.

Dr. Lippe, in answer to Dr. W. M. James, said : In my paper (on the Repetition of the Dose) this evening, I wish the inference to be drawn that a remedy may not show its action for three days after taking it. Therefore we must wait. Dr. Jacob Jeanes, who was a very acute prover, used to say that when he took a remedy for proving, he could tell within a minute if it were going to act, by some slight symptom. Then it would lie quiet for three days before he could get symptoms.

Dr. James asked for a remedy having cough caused by tickling, with tickling increased by coughing, and extending up into the ears.

Dr. Lippe answered that Cistus can. is a remedy having this symptom.

Geo. H. Clark.

[Ignatia: The longer he coughs, the more the irritation to cough increases. Marum Verum Teucrium: Short, dry cough from tickling in upper part of trachea aggravated by coughing. See Lippe's *Materia Medica.*—EDS.]

